# High-Efficiency and Narrowband Green Thermally Activated Delayed Fluorescence Organic Light-Emitting Diodes Based on Two Diverse Boron Multi-Resonant Skeletons

**DOI:** 10.3390/molecules29040841

**Published:** 2024-02-14

**Authors:** Zhen Wang, Cheng Qu, Jie Liang, Xuming Zhuang, Yu Liu, Yue Wang

**Affiliations:** 1 State Key Laboratory of Supramolecular Structure and Materials, College of Chemistry, Jilin University, Changchun 130012, China; zwang21@mails.jlu.edu.cn (Z.W.); qucheng20@mails.jlu.edu.cn (C.Q.); yuewang@jlu.edu.cn (Y.W.); 2 Jihua Laboratory, 28 Huandao South Road, Foshan 528200, China; liangjie@jihualab.com (J.L.); zhuangxm@jihualab.com (X.Z.)

**Keywords:** organic light-emitting diodes (OLEDs), thermally activated delayed fluorescence (TADF), oxygen-bridged boron, multiple resonance (MR), narrowband

## Abstract

Up to now, highly efficient narrowband thermally activated delayed fluorescence (TADF) molecules constructed by oxygen-bridged boron with an enhancing multiple resonance (MR) effect have been in urgent demand for solid-state lighting and full-color displays. In this work, a novel MR-TADF molecule, BNBO, constructed by the oxygen-bridged boron unit and boron–nitrogen core skeleton as an electron-donating moiety, is successfully designed and synthesized via a facile one-step synthesis. Based on BNBO as an efficient green emitter, the organic light-emitting diode (OLED) shows a sharp emission peak of 508 nm with a full-width at half-maximum (FWHM) of 36 nm and realizes quite high peak efficiency values, including an external quantum efficiency (EQE_max_) of 24.3% and a power efficiency (PE_max_) of 62.3 lm/W. BNBO possesses the intramolecular charge transfer (ICT) property of donor-acceptor (D-A) materials and multiple resonance characteristics, which provide a simple strategy for narrowband oxygen–boron materials.

## 1. Introduction

As the third-generation organic electroluminescent emitter for organic light-emitting diodes (OLEDs), the thermally activated delayed fluorescence (TADF) material system has made considerable progress in the past decade due to its possible commercial application in large flat panel displays and solid-state lighting. Although TADF luminescent materials are the typical organic small molecules without heavy metal atoms, they can achieve 100% internal quantum efficiency through reverse intersystem crossing (RISC). The triplet states can also participate in the emission as delayed fluorescence through the fast RISC process [1,2]. Various strategies have been employed to expedite the RISC process in TADF materials by reducing the singlet–triplet (S1-T1) energy gaps. These approaches include the design of a large dihedral angle in donor-acceptor-type (D-A) systems. While such designs promote highly efficient TADF emission, they often lead to increased structural and vibrational relaxation in the S1 state and between the ground state (S0) and S1 state. As a consequence, this can result in a broad emission band with diminished color purity and elevated nonradiative decay rates [3,4,5,6]. In addition, one more pivotal issue of this strategy is the decrease in oscillator strength (f), leading to lower photoluminescence quantum yields (PLQYs, ΦPL), which is not conducive to achieving a high device efficiency [7,8].

Recently, due to the minimization of both bonding and antibonding characteristics between adjacent atoms and the resulting induced HOMO-LUMO separation [6,7,9,10,11], a series of unprecedented TADF materials that can utilize the multiple resonance (MR) effect to exhibit narrow emissions and be employed as high-efficiency OLED materials have been reported, where the Franck–Condon excitations show a high oscillator strength reminiscent of locally excited (LE) states and short-range charge transfer (SRCT) states, leading to both efficient delayed fluorescence and a high ΦPL. Owing to MR effects, multitudinous boron-nitrogen (B-N) types, boron-oxygen (B-O) types, and other specific types, such as nitrogen–carbonyl-type emitters, exhibit excellent device performances with typical small full-width at half-maximums (FWHMs) [12,13,14,15,16], especially for the green and blue emitters based on B-N core skeletons with donor units [17,18,19,20]. Alternatively, in addition, B-O emitters also exhibited a breakthrough performance, and oxygen-bridged boron cores are employed as the acceptor unit with rigid and symmetric structures [21,22,23,24,25,26]. Thus far, high-efficiency B-O emitters’ colors have been achieved in full-color spectral regions combined with various donor units, whereas most B-O molecules reported are facing the problems of peak width broadening and color purity reduction caused by the enhancement of long-range CT states, especially for green or red materials. In relation to the D-A structure, combining it with strong donor units could achieve redshifted emission, which, however, would reduce the intensity of the MR effect of the whole molecules. Upon expanding the conjugation of B-O materials or the stronger electron-donating strength of donor units, the MR structure will be destroyed or only restricted to the oxygen-bridged oxygen core segments. Therefore, the construction of narrowband green and red emissive materials based on B-O core skeletons remains a crucial challenge.

To tackle the issue of broadening emission bands, the nitrogen–carbonyl core is combined with the boron-oxygen core to achieve a narrowband blue emitter (BOQAO), which shows weak long-range CT states and an EQE performance of 21.8% with an FWHM of 32 nm [27]. In contrast to the nitrogen–carbonyl core, B-N-type cores can be employed as better and stronger donor units with weaker long-range CT states and more effective short-range CT states, such as the DtBuCzB core. Thus, considering a similar strategy, the introduction of MR cores instead of conventional donor units will improve the broadened emission from the strong CT states. Therefore, the combination with rigid MR cores can be the practical pathway for the narrowband green B-O materials. Herein, we synthesized and characterized a novel MR-TADF emitter, BNBO, based on two diverse boron multi-resonant skeletons, boron-oxygen and boron-nitrogen cores. Different from the conventional D-A-type TADF, the B-N segment exhibits both electron-donating and electron-withdrawing abilities, and it can provide a sufficient donor capacity for redshifted emission. Through bonding with the B-N segment, the boron-oxygen with acceptor characteristics forms a weak CT state with it, resulting in a redshift from violet to green emissions. Furthermore, the multiple resonance effect and planar rigidity of the boron-nitrogen structure significantly facilitate the highly efficient narrowband emission of BNBO. These are the desirable advantages of selecting the boron-nitrogen group to construct novel boron-oxygen emitting molecules. As we expected, the organic light-emitting diode (OLED) based on BNBO as an efficient green emitter shows a sharp emission peak of 508 nm with a full-width at half-maximum (FWHM) of 36 nm and realizes quite high peak efficiency values, including an external quantum efficiency (EQE_max_) of 24.3% and a power efficiency (PE_max_) of 62.3 lm/W, indicating that this is an effective approach to further enrich narrow-emission-band emitting systems.

## 2. Results and Discussion

### 2.1. Synthesis and Characterization

BOBr (501 mg, 1.08 mmol), BNpin (870 mg, 1.13 mmol), potassium carbonate (414 mg, 3.00 mmol), water (2 mL), and THF (15 mL) were added to a 50 mL two-necked flask. The mixture was bubbled with nitrogen for a further 10 min, and tetrakis(triphenylphosphine)palladium(0) (39.2 mg, 0.034 mmol) was added under a high flow of nitrogen. Then, the mixture was heated to reflux and stirred overnight. After cooling it to room temperature and adding water and dichloromethane, the organic layer was washed with saturated brine twice, collected, and condensed in vacuo. The crude product was further purified using column chromatography to obtain the target compound (BNBO) as a bright yellow solid: (0.6 g, 54%) ^1^H NMR (500 MHz, Methylene Chloride-*d*_2_) δ 9.16 (d, *J* = 1.8 Hz, 2H), 8.84 (d, *J* = 2.5 Hz, 2H), 8.70 (s, 2H), 8.55 (d, *J* = 7.5 Hz, 4H), 8.34 (d, *J* = 2.0 Hz, 2H), 7.87 (dd, *J* = 8.7, 2.4 Hz, 2H), 7.84 (d, *J* = 1.5 Hz, 2H), 7.78 (dd, *J* = 8.6, 2.0 Hz, 2H), 7.61 (d, *J* = 8.7 Hz, 2H), 1.72 (s, 18H), 1.58 (s, 21H), 1.56 (s, 18H). ^13^C NMR (500 MHz, Methylene Chloride-*d*_2_) δ 158.71, 157.92, 145.32, 145.07, 144.55, 138.10, 131.50, 130.21, 129.63, 126.98, 124.51, 123.55, 121.65, 121.48, 120.80, 117.78, 117.30, 114.18, 107.50, 107.11, 35.04, 34.68, 34.44, 31.92, 31.58, 31.32. The relevant NMR spectra can be found in Figures S1 and S2 (ESI file). ESI-MS (M): *m*/*z*: 1020.77 [M^+^]+ (calcd: 1020.59). Anal. Calcd for C_72_H_74_B_2_N_2_O_2:_ C, 84.70; H, 7.31; N, 2.74; Found: C, 84.48; H, 7.37; N. 2.72.

**Scheme 1:**
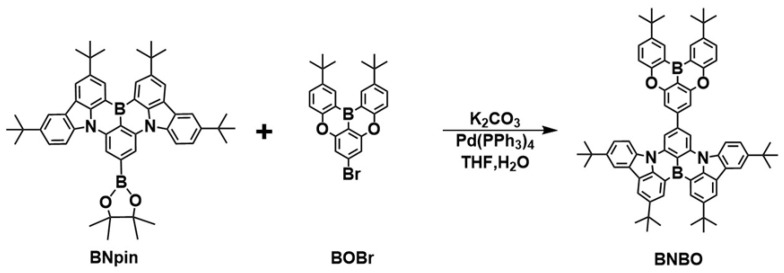
Synthetic procedures of BNBO.

**Figure 1:**
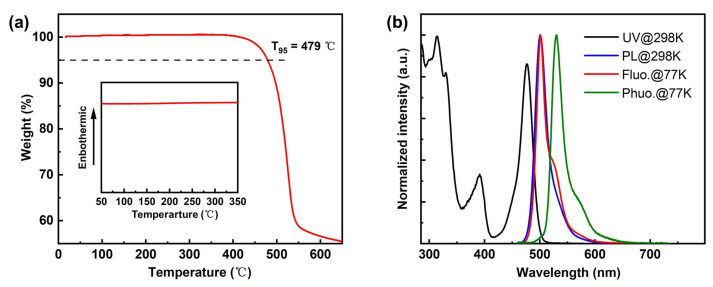
(**a**) TGA and DSC (inset) diagrams of BNBO; (**b**) ultraviolet–visible (UV–vis) absorption/photoluminescence (PL) spectra at room temperature (298 K) and fluorescence/phosphorescence spectra at low temperature (77 K) of BNBO measured in toluene solutions (10^−5^ M).

By connecting the planar and rigid B-N core (DtBuCzB), which has been reported as being used as the significant main core for the construction of diverse B-N narrowband emitters for understanding the MR effect and high EQEs in devices [28], with the weak boron acceptor (B-O core) at the para-position, this novel green MR-TADF BNBO is synthesized, where the strong electron-donating ability of the B-N core segment is expected to endow the emitter BNBO with strong intramolecular charge transfer (ICT) characteristics, thereby leading to redshifted wavelength emission. The simple one-step synthesis of BNBO is shown in Scheme 1. BNpin is reported as a significant synthetic intermediate to obtain diverse boron-nitrogen-type MR-TADFs [29]. The key compounds, BOBr and BNpin, are prepared from commercially available starting materials according to the previous literature [22,29]. The target compound BNBO is prepared from one simple Suzuki coupling reaction between BNpin and BOBr in a very high yield. The molecular structure of the synthesized molecule is fully confirmed via proton (^1^H) nuclear magnetic resonance (NMR) spectroscopy, carbon (^13^C) NMR spectroscopy, elemental analysis, and mass spectrometry. The thermal and morphological stabilities are also examined via thermogravimetric analysis (TGA) and differential scanning calorimetry (DSC) (Figure 1a). Benefiting from the highly twisted structure and rigid scaffold of MR cores, the high decomposition temperature (T_d_) at which a 5% weight loss occurs upon heating is measured to be 479 °C, which suggests its excellent thermal stability. In addition, no apparent glass transition temperature (T_g_) is found in the DSC curve; this, together with the sufficiently high T_d_ and undetectable T_g_, indicates that the target compound is suitably thermally stable to be considered as a candidate for OLED fabrication.

### 2.2. Photophysical and Electrochemical Properties

As illustrated in Figure 1b and Table 1, BNBO exhibits intense and sharp absorption bands from 315 to 477 nm, with the maximum absorption peak at 315 nm from the π-π* transitions. Moreover, similar to the reported boron-nitrogen materials, another strong absorption at 477 nm is associated with the ICT transition, which is attributed to the strong short-range CT states. This compound displays green emission in toluene solution with the maximum fluorescence spectrum peaking at 500 nm with a small Stokes shift at 23 nm as well as a quite narrow FWHM of 25 nm. Notably, the UV–vis absorption and PL spectral profiles exhibit the typical MR-type shaped mirror symmetry, and the area of overlap between the absorption and emission spectrum is minimal, which shows the ICT characteristic of the excited states. The existence of two rigid core skeletons contributed to a slight excited-state structural relaxation and weak LRCT states, inducing an exceedingly small Stokes shift, and the S_1_ and T_1_ energies of BNBO can be determined from the onset of the low-temperature photoluminescence spectra, whose values are 2.55 eV and 2.41 eV, respectively, as shown in Figure 1b. The corresponding Δ*E*_st_ value of 0.14 eV is theoretically tiny enough to promise the exciton up-conversion avenue with ambient thermal energy to promote the RISC process. In addition, the low-temperature fluorescence spectrum was almost identical to the fluorescence spectrum at room temperature, and the shoulder peak at 526 nm was slightly enhanced, reflecting the existence of internal vibration.

**Figure 2:**
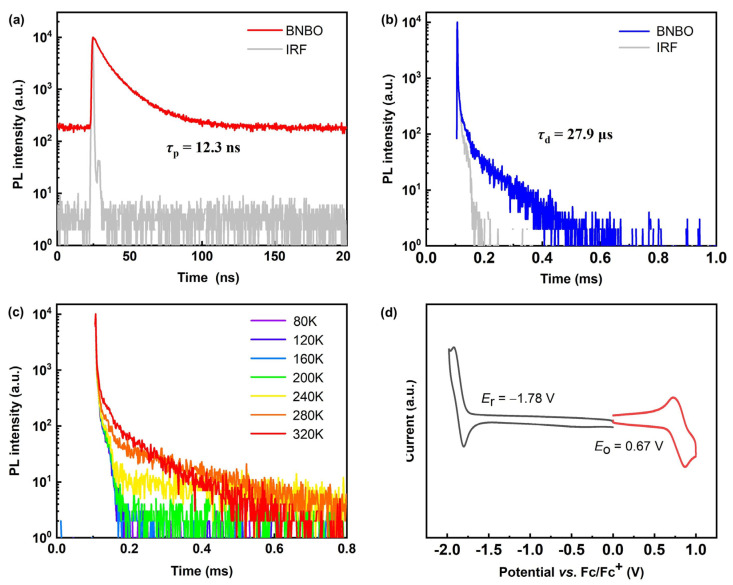
(**a**) Prompt fluorescence lifetime curves and (**b**) delayed fluorescence lifetime curves and (**c**) normalized temperature-dependent PL intensity decay curves of the doping deposited film based on BNBO in PhCzBCz with 5 wt% concentration; (**d**) CV curves of BNBO.

Further, the TADF nature can be demonstrated and confirmed by the transient photoluminescence of the vacuum-evaporated doped films of PhCzBCz (9-(2-(9-phenyl-9*H*-carbazol-3-yl)phenyl)-9*H*-3,9′-bicarbazole): BNBO with a concentration of 5 wt%, which showed distinct short-lived prompt fluorescence and long-lived delayed fluorescence properties, respectively, as shown in Figure 2a,b. Moreover, as illustrated in Figure 2c, when the ambient temperature was increased from 80 K to 320 K, the ratio of the delayed component from the recursive S_1_ → S_0_ transition via the accelerated up-conversion of triplet excitons enhanced gradually, thus proving the typical TADF property. Prompt fluorescence with a lifetime *τ*_PF_ of 12.3 ns and delayed fluorescence with a lifetime (*τ*_DF_) of 27.9 μs shorter than reported boron-nitrogen materials were detected in the transient fluorescence decay measurements, and the radiative decay rate constant (k_ic_), intersystem crossing rate constant (k_ISC_), and RISC rate constant (k_RISC_), which are 3.58 × 10^8^ s^−1^, 4.51 × 10^8^ s^−1^, and 3.46 × 10^4^ s^−1^, respectively, were also calculated. The fast rate of the reverse intersystem crossing is associated with the small ∆*E*_ST_ and the short *τ*_DF_, and the rate constant of the RISC (*k*_RISC_) is large enough for the efficient triplet exciton up-conversion. Notably, the large radiation transition rate constant (k_r_) of BNBO, which is as large as 10^8^ s^−1^ orders of magnitude, significantly surpasses the nonradiative decay rate constants (k_nr_), in agreement with the large *f* value and tiny structural relaxation. The calculated SOC value <S_1_|*H*_SOC_|T_2_> (0.127 cm^−1^) of BNBO is much larger than <S_1_|*H*_SOC_|T_1_> (0.0142 cm^−1^), indicating that the RISC process is partly dominated by the T_2_ state to S_1_ state. Thus, the energetic closeness in terms of S_1_ and triplet states (T_1_ and T_2_) is beneficial for facilitating the RISC and the full utilization of triplet excitons.

The HOMO and LUMO energy levels and electrochemistry properties of BNBO were determined using cyclic voltammetry (CV), as shown in Figure 2d and Table 1. The HOMO and LUMO levels were calculated from the onset of the oxidation and reduction potentials, and their values were −5.13 eV and −2.73 eV, respectively, in good consistency with the DFT results. The oxidation peak potential corresponding to the HOMO is relatively close to the reported boron-nitrogen core, which is in full agreement with the theoretical calculation results. It can be known from the reversible redox peak that BNBO has good electrochemical stability. Hence, BNBO is suitable for the fabrication of stable and excellent OLEDs.

### 2.3. Theoretical Calculation

**Figure 3:**
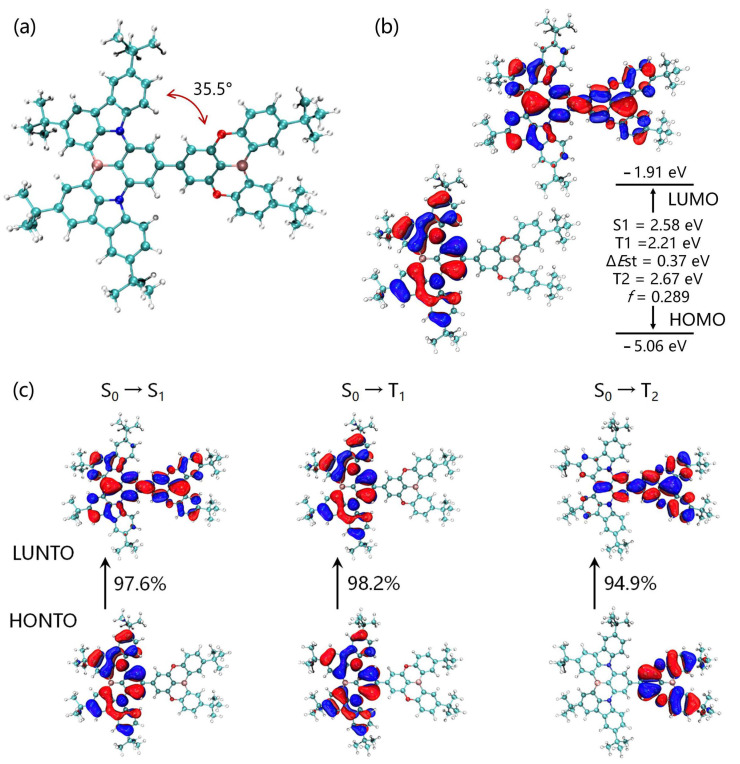
(**a**) Optimized ground-state configuration and dihedral angle of BNBO; (**b**) HOMO/LUMO distributions, excited-state energy levels, and oscillator strength of BNBO; (**c**) natural transition orbital calculation for excited states hole (blue) and electron (white) for S_1_, T_1_ and T_2_.

Density functional theory (DFT) and time-dependent density functional theory (TDDFT) simulations were used to gain deep insights into the geometrical and electronic structure of BNBO, including the frontier molecular orbital (FMO) distributions. This compound has a warped molecular configuration with a moderate dihedral angle of 35.5° between the boron-nitrogen core segment and the oxygen-bridged boron moiety (Figure 3a), which was insufficient for the spatial separation of molecular orbitals in terms of conventional D-A-type materials. As shown in Figure 3b, as in our prediction, the HOMOs are utterly distributed on the boron-nitrogen core skeleton, which fully reflects its strong donor property, and the LUMOs are distributed entirely on the molecular framework rather than the boron-oxygen core.

The corresponding HOMO and LUMO energy levels of BNBO were calculated to be −5.06 eV and −1.91 eV with a bandgap value of 3.15 eV. Furthermore, BNBO shows typical MR characteristics on the entire molecular skeleton, which indicates its higher PLQY and larger oscillator strength than the simple D-A boron-oxygen core moiety with ICT. More than this, the FMOs are present on the B-N core skeleton and display alternating increasing and decreasing densities on adjacent sites. This suggests the existence of a short-range charge transfer (SRCT) and then ensures a suitably small Δ*E*_st_ to enable an efficient RISC for TADF. In addition, the LUMOs of the B-O core segment suggest a long-range charge transfer (LRCT) between the B-O component and the B-N core moiety; thus, the fluorescence emission mainly occurs from adequate ICT states composed of an LRCT and SRCT. Therefore, BNBO shows promise to achieve the target narrowband TADF emission compared with some cases based on boron-oxygen materials connecting with some conventional donors such as diphenylamine.

To further investigate the excited-state electronic properties of BNBO exhaustively, natural transition orbital (NTO) analysis of BNBO for the transition to the singlet excited state (S_1_) and the triplet excited state (T_1_/T_2_) was also carried out and is depicted in Figure 3c. Similar to the ground state (S_0_), the highest occupied NTOs (HONTOs) and the lowest unoccupied NTOs (LUNTOs) are located on the boron-nitrogen core skeleton and all parts of BNBO, respectively. For the S_0_ → S_1_ transition, the HONTOs and LUNTOs are identical to its corresponding HOMO and LUMO of S_0_, suggesting the singlet emission can be explained as a mixed transition consisting of locally excited (LE) and charge transfer (CT) state characteristics in the S_1_ state. For the S_0_ → T_1_ transition, the LUNTOs and HONTOs of BNBO are entirely located on the boron-nitrogen core segment, indicating the LE emission. Notably, the LE characteristics in triplet states are vital in boosting the RISC process by enhancing the spin-orbit coupling (SOC) between the S_1_ state with CT characteristics and triplet states. Normally, the S_1_ and T_1_ NTOs shared a similar spatial region, which is favorable for the ease of RISC processes, leading to an increased RISC rate. Generally, the structural variations between S_0_ and S_1_ are closely related to the FWHMs of the emission spectra. The above results indicated that introducing boron-nitrogen core segments to the oxygen-bridged boron core could effectively suppress the molecular configuration relaxation of the radiative transition process and maintain the MR luminescence characteristic as well as strong ICT states.

### 2.4. Electroluminescence Properties

**Figure 4:**
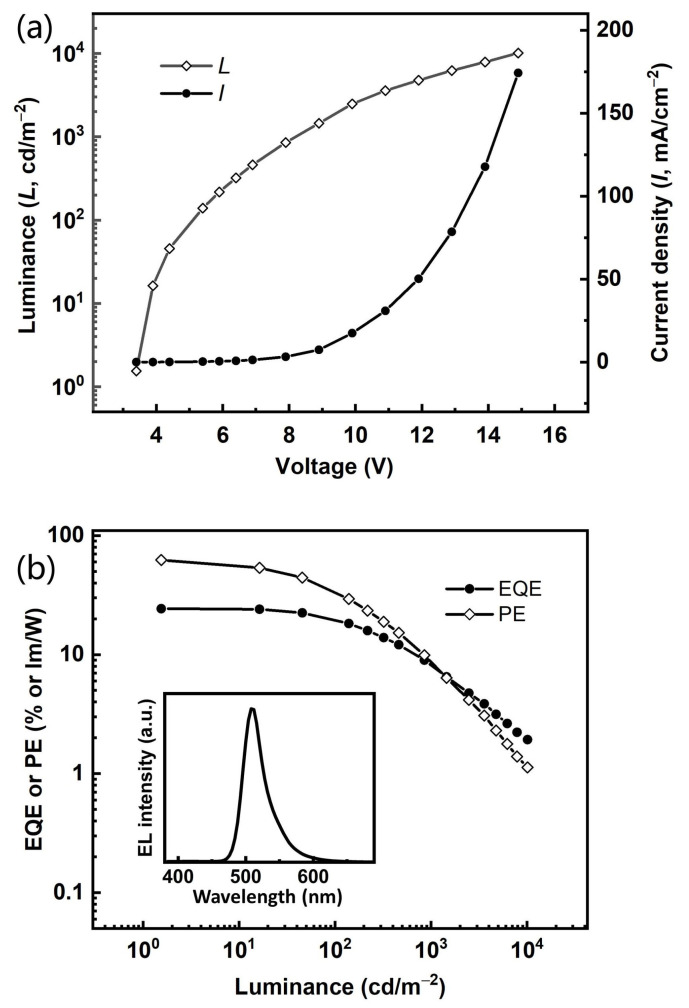
(**a**) *J*–*V*–*L* characteristics and (**b**) EQE-PE–*L* characteristics of device (inset: EL spectra of device at a luminance of 100 cd/m^−2^ and a current density of 0.35 mA/cm^−2^ under an applied voltage of 5.2 V).

Based on the excellent narrowband spectral curve and the desirable photophysical characteristics, this TADF compound BNBO‘s relevant properties as an electroluminescent emitter should be further investigated. Thus, a vacuum-evaporation OLED with emitting layers (EML) made through doping BNBO into PhCzBCz with a 5% concentration is fabricated by adopting the following configuration: ITO/TAPC (50 nm)/TCTA (5 nm)/PhCzBCz: 5 wt% emitters (30 nm)/TmPyPB (30 nm)/LiF (1.5 nm)/Al (100 nm), where ITO (indium tin oxide) and Al (aluminum) were used as the anode and cathode, respectively. TAPC (1,1-bis [4-[*N*, *N*-di(p-tolyl)amino] phenyl]cyclohexane), TmPyPb (1,3,5-tri(mpyrid-3-yl-phenyl)benzene), and LiF functioned as the hole transporting layer (HTL), electron transporting layer (ETL), and electron injecting layer (EIL), respectively. To prevent excitons from diffusing into the TAPC layer, we employed TCTA (tris(4-carbazolyl-9-ylphenyl)amine) as an exciton blocking layer (EBL) due to its high triplet energy level. PhCzBCz was chosen as the rigid host with the fast hole-transporting ability to form the emitting layer with a good orbital energy alignment, which can accelerate the RISC and ISC processes synchronously. The energy transfer and transfer involve injecting holes and electrons from the anode and cathode, respectively, which traverse the transport layers to reach the central emissive layer for recombination, resulting in exciton generation. The correlative energy diagrams of the OLEDs, the molecular structures of the materials used in devices, and the relevant EL characteristics are illustrated in Figure S4 (Supplementary Materials) and Figure 4, and the detailed device data are summarized in Table 2.

This device maintains green emission with a sharp peak at 508 nm with a modest FWHM of 36 nm), which is considerably the smallest FWHM among the reported boron-oxygen derivatives in the green region, and CIE coordinates of (0.17, 0.65. It displays marginally bathochromic-shifted emissions with their respective PL spectrum in dilute solution. No host emission is observed in the EL spectrum, demonstrating that the radiative transition excitons are well confined in the EML and the energy transfer from host to dopant emitter should be complete. It exhibits a quite low turn-on voltage of 3.4 V, which demonstrates the efficient carrier injection and transporting in the whole emitting system, and the maximum EQE and power efficiency (PE) values are 24.3% and 62.3 lm/W, respectively. They can maintain quite high levels of 20.8% and 34.6 lm/W at 100 cd m^−2^, indicating that the exciton concentration quenching at high voltages has been inhibited, which should be a benefit from the relatively short delayed lifetime (27.9 μs) of this compound. Such a desirable EL performance represents one of the best green narrowband emitting systems among those based on the skeletons containing the boron-oxygen and boron-nitrogen cores, which principally originates from the fast RISC process, the high fluorescent radiative efficiency of the transition from S_1_ → S_0_, and the high *f* value. The comprehensive performance of the green BNBO device, including remarkable EQEs and small FWHMs, convincingly demonstrates that the molecular design approach for constructing narrowband green B-O materials is feasible and efficient.

## 3. Materials and Methods

### 3.1. General Information

Organic solvent and materials obtained from commercial suppliers were used without further refinement. The round-bottom flask, magnetic stirring bars, and syringe needles were dried in an oven. All the reactions dealing with air- or moisture-sensitive compounds were carried out in a dry reaction vessel under positive nitrogen pressure. Reactions were monitored with thin layer chromatography (TLC) at 254 nm and 365 nm UV light. The MALDI MS dates were collected using an Autoflex speed TOF/TOF mass spectrometer (Bruker Daltonics, Bremen, Germany) with a pulsed Nd:YAG laser (355 nm). A flash EA 1112 spectrometer (Elementar, Langenselbold, Germany) was used to perform the elemental analyses. Bruker AVANCE III 500 MHz spectrometers (Bruker, Bremen, Germany) were selected to measure the ^1^H and ^13^C NMR spectra, respectively, with tetramethylsilane (TMS) as the internal standard. Infrared spectra (IR) were obtained using the Thermo Nicolet iS50 instrument (Thermo Fisher Scientific, Waltham, MA, USA). In the range of 25 to 650 °C, a TA Q500 thermogravimeter (TA, New Castle, DE, USA) was selected to perform the thermogravimetric analysis (TGA) of target molecules under a nitrogen atmosphere at a heating rate of 10 K min^–1^. Differential scanning calorimetric (DSC) measurement was performed on a NETZSCH DSC204 instrument (NETZSCH, Selb, Germany) at a heating rate of 10 K min^–1^ under a nitrogen atmosphere.

### 3.2. Photophysical Characterizations

A Shimadzu RF-5301 PC spectrometer (Shimadzu, Tokyo, Japan) and Shimadzu UV-3600 spectrophotometer (Shimadzu, Tokyo, Japan) were adopted to record the PL emission spectra and UV-Vis absorption, respectively. The fluorescence and phosphorescent spectra taken at liquid nitrogen temperature (77 K) were recorded using an Ocean Optics QE Pro with a 365 nm Ocean Optics LLS (Ocean Optics, Orlando, FL, USA) excitation source. An Edinburgh FLS920 steady-state fluorimeter (Edinburg, Livingston, UK) equipped with an integrating sphere was employed to measure the absolute fluorescence quantum yields of doped films. An FLS980 fluorescence lifetime measurement system (Edinburg, Livingston, UK) with a 365 nm LED excitation source was selected to investigate the transient PL decay.

### 3.3. Cyclic Voltammetry

A BAS 100 W Bioanalytical electrochemical workstation (BAS, West Lafayette, IN, USA) was used to measure the electrochemical properties with a platinum disk as the working electrode, platinum wire as the auxiliary electrode, a porous glass wick Ag/Ag^+^ as a pseudo reference electrode, and ferrocene/ferrocenium as the internal standard. And a 0.1 M solution of nBu4NPF6, the supporting electrolyte, was utilized to measure the oxidation (in anhydrous CH_2_Cl_2_) or reduction (in anhydrous tetrahydrofuran) potentials at a scan rate of 100 mV s^−1^.

### 3.4. Device Fabrication and Measurements

Before the device fabrication, the indium tin oxide (ITO) glass substrates with a sheet resistance of 15 Ω per square were treated with plasma for 5 min, after cleaning with optical detergents, deionized water, acetone, and isopropanol successively. Then, they were transferred to a vacuum chamber in a nitrogen-filled glove box. In the vacuum chamber, there were various evaporation sources for evaporating organic and inorganic materials, and the upper ITO glass substrate rotated at a consistent speed around the central axis of the vacuum chamber. Once a high vacuum level (<9 × 10^−5^ Pa) was achieved, the organic materials were deposited onto the ITO glass substrates at a rate of 1 Å s^−1^. Real-time monitoring and control were implemented using a centrally fixed quartz-crystal thickness monitor. The emitting layer composed of multiple components was carefully monitored with dual probes to maintain a constant evaporation rate ratio, which ensured that the concentration of the doped guest remained unchanged. After finishing the deposition of organic layers, ITO glass substrates were patterned by a shadow mask with an array of 2.0 mm × 2.5 mm openings. Then, LiF and Al were successively deposited at rates of 0.1 Å s^−1^ and 5 Å s^−1^, respectively. The electrical characteristics of the OLEDs were determined using a Keithley 2400 source meter (Keithley, Cleveland, OH, USA). The EL spectra and luminance of the OLED devices were recorded on a PR650 spectrometer (Photo Research, Syracuse, NY, USA). All measurements were taken on the devices without encapsulation in an ambient atmosphere in the dark.

## 4. Conclusions

In conclusion, a representative rare molecular structure paradigm based on a B-O-embedded B-N framework has been successfully obtained and applied to an efficient OLED being utilized as an emitter, which displays green emission with a peak of 508 nm, a narrow FWHM of 36 nm, CIE coordinates of (0.17, 0.65), a maximum EQE of 24.3%, and a low efficiency roll-off. The target model, BNBO, not only inherits the inherent narrowband emission curve of the B-N-MR skeleton but also retains the striking advantages of a short lifetime and low-efficiency roll-off that originated from the inherent rigid and symmetric structures owned by the B-O-core. The excellent EL performances of BNBO have constructed a pragmatic and heuristic molecular model of material research for an ultrahigh-definition display. Most importantly, this successful design concept establishes a concrete molecular structure paradigm and provides an effective strategy for the construction of new TADF emitters with narrowband emission towards an ultrahigh-definition display.

## Figures and Tables

**Table 1:** Summary of photophysical and electrochemistry properties of BNBO.

*λ*_abs_ ^a^ [nm]	*λ*_em_ ^b^ [nm]	FWHM ^c^ [nm]	Δ*E*_st_ [eV]	*τ*^d^ [ns]	*τ*^e^ [µs]	HOMO/LUMO ^f^ [eV]
315,330,391,477	500	25	0.14	12.3	27.9	−5.13/−2.73

^a^ Peak wavelength of absorption peak in toluene solution (1 × 10^−5^ M, 298 K). ^b^ Peak wavelength of PL spectrum in toluene solution (1 × 10^−5^ M, 298 K). ^c^ Full-width at half-maximum. ^d^ Lifetime of prompt fluorescence and ^e^ lifetime of delayed fluorescence of the doping deposited film based on BNBO in PhCzBCz with 5 wt% concentration. ^f^ Determined from cyclic voltammetry using the following formula: *E*_HOMO_ = − (*E*_ox_ + 4.8) eV and *E*_LUMO_ = − (*E*_red_ + 4.8) eV.

**Table 2:** Summary of the EL data of device.

λ ^a^ [nm]	FWHM ^b^ [nm]	CIE ^c^	V_turn-on_^d^ [V]	L_max_ [cd/m^2^]	PE ^e^ [lm/W]	EQE ^e^ [%]	Roll-Off ^f^ Ratio [%]
508	36	(0.17, 0.65)	3.4	10,100	62.3/34.6	24.3/20.8	14.5

^a^ EL peak wavelength at 100 cd/m^2^. ^b^ Full-width at half-maximum. ^c^ Commission Internationale de L’Eclairage coordinates (value taken at 100 cd/m^2^). ^d^ Turn-on voltage at 1 cd/m^2^. ^e^ Maximum efficiencies and the values at 100 cd/m^2^, respectively. ^f^ Efficiency roll-off ratio (EQE at 100 cd/m^2^ relative to EQE_max_).

## Data Availability

Data are contained within the article and supplementary materials.

## Supplementary Materials

The following supporting information can be downloaded at: https://www.mdpi.com/article/10.3390/molecules29040841/s1. Figure S1: ^1^H NMR spectrum of BNBO in CD_2_Cl_2_; Figure S2: ^13^C NMR spectrum of BNBO in CD_2_Cl_2_; Figure S3: Infrared spectrum of BNBO; Figure S4: Molecular structure of device-related materials and device structure, schematic diagram of mechanism.
