# Supplementary material for: High-Efficiency and Narrowband Green Thermally Activated Delayed Fluorescence Organic Light-Emitting Diodes Based on Two Diverse Boron Multi-Resonant Skeletons
Source: Molecules. 2024 Feb 14;29(4):841. doi: 10.3390/molecules29040841 (PMC10892125; doi:10.3390/molecules29040841)
Supplement: Supplementary file 1 [file molecules-29-00841-s001.zip › molecules-2841942-supplementary.pdf]

Supporting Information

High-efficiency and narrowband green thermally activated de-layed fluorescence organic light-emitting diodes based on two diverse boron multi-resonant skeletons

Zhen Wang[1], Cheng Qu[1], Jie Liang[2], Xuming Zhuang[2], Yu Liu[1,*] and Yue Wang[1]

1 State Key Laboratory of Supramolecular Structure and Materials, College of Chemistry, Jilin University, Changchun 130012, P. R. China

2 Jihua Laboratory, 28 Huandao South Road, Foshan, 528200, Guangdong Province, P. R. China

* Correspondence: yuliu@jlu.edu.cn (Y.L)

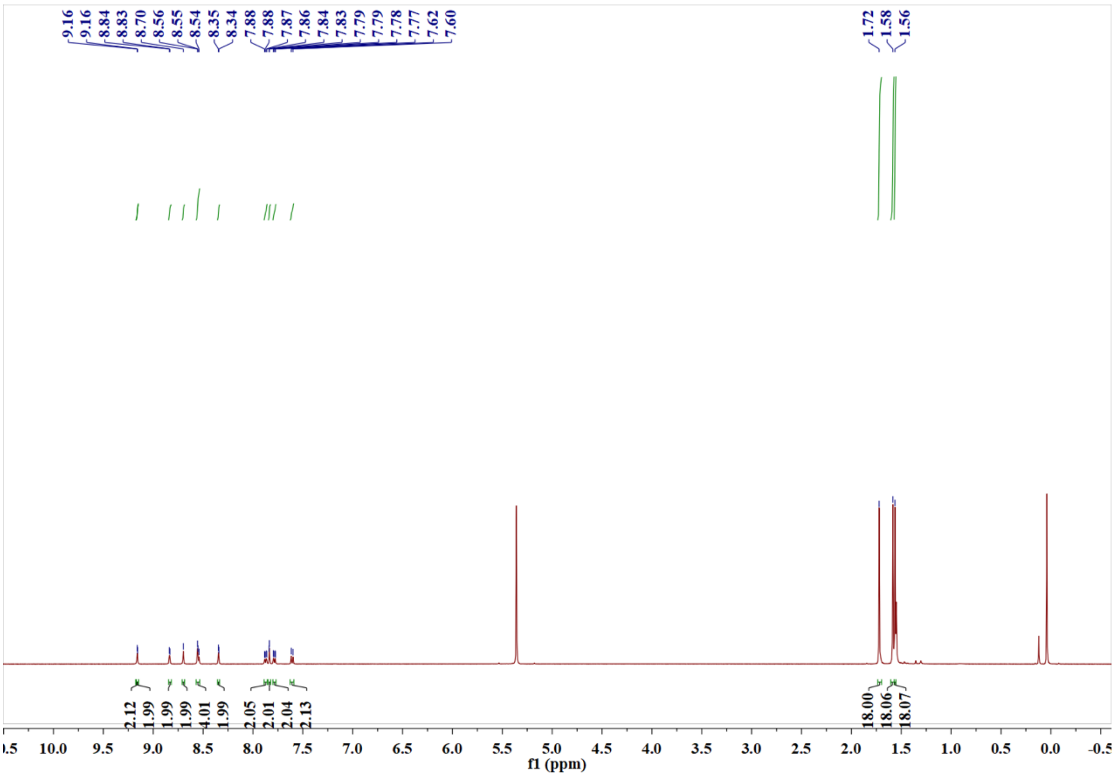

Figure S1. ^{1}H NMR spectrum BNBO (500 MHz, Methylene Chloride-d_2)

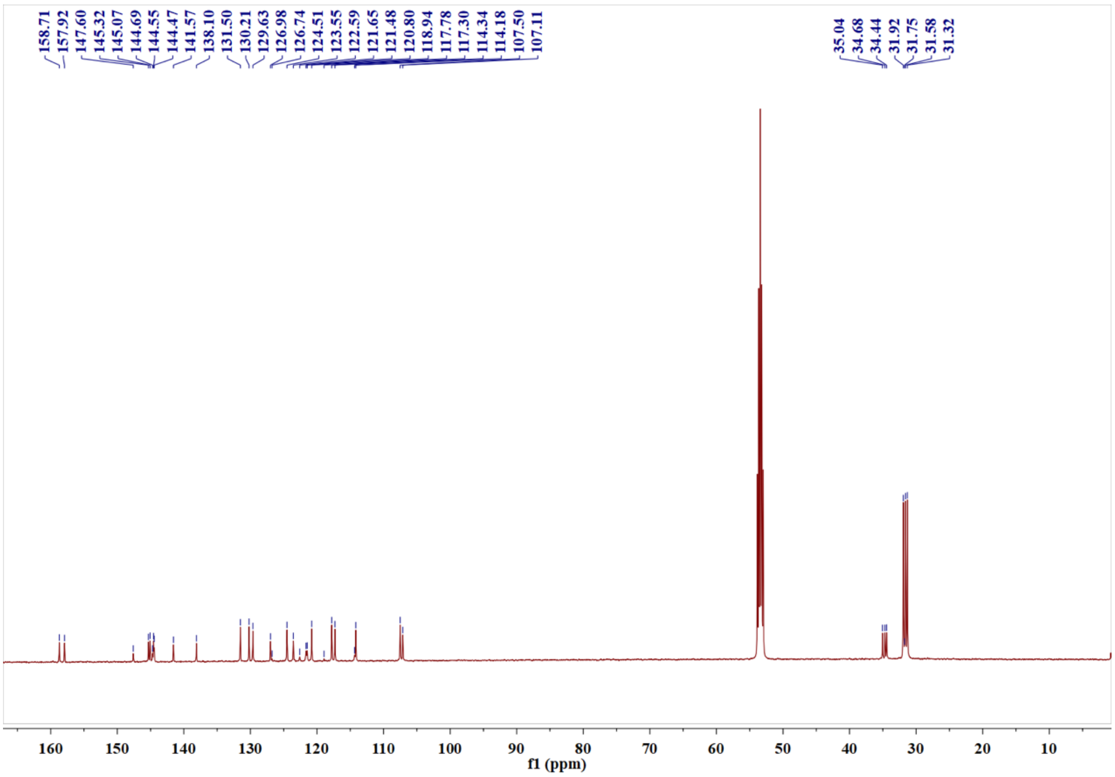

Figure S2. ^{13}C NMR spectrum BNBO (500 MHz, Methylene Chloride-d_2)

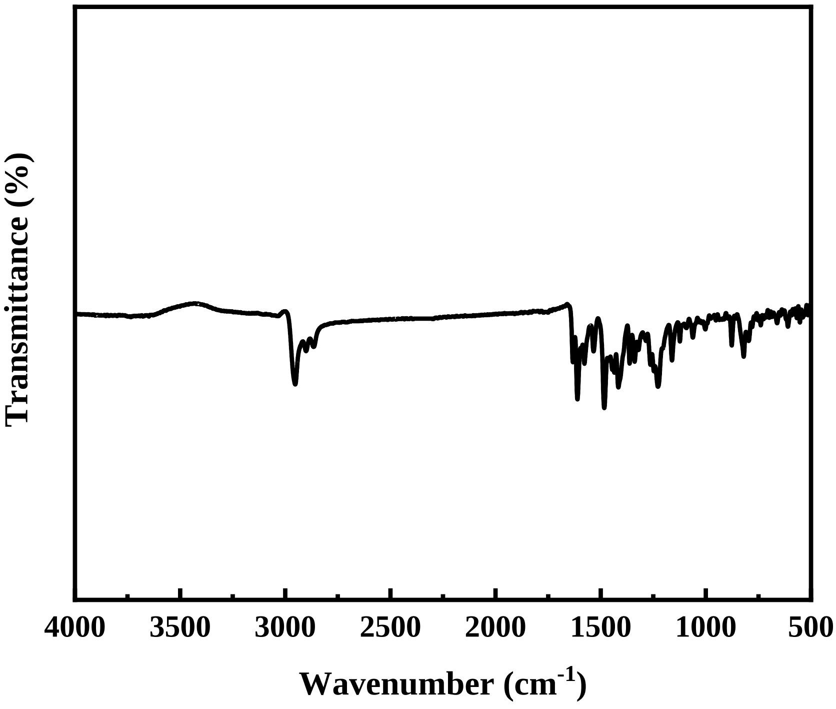

Figure S3. Infrared spectrum of BNBO

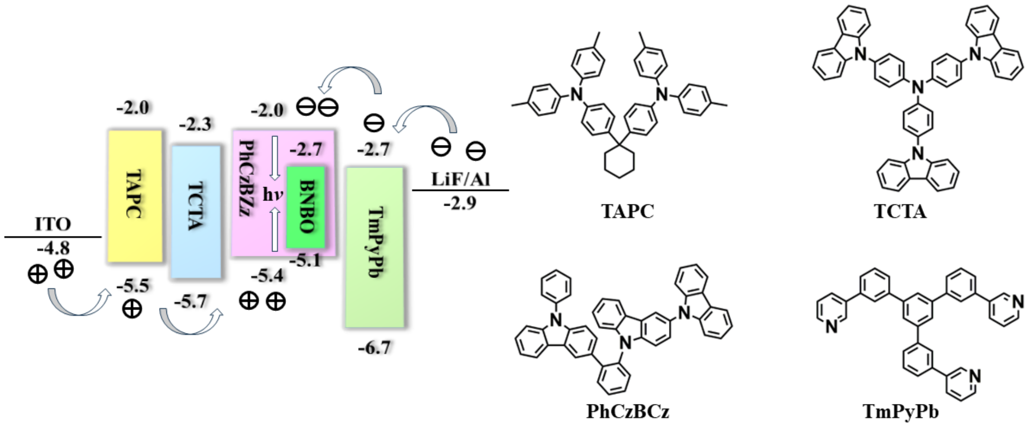

Figure S4. Molecular structure, energy level structure and schematic diagram of electroluminescence mechanism for device
